# Help for Future Research: Lessons Learned in Trial Design, Recruitment, and Delivery From the “hELP” Study

**DOI:** 10.1097/LGT.0000000000000407

**Published:** 2018-07-02

**Authors:** Rosalind C. Simpson, Ruth Murphy, Daniel J. Bratton, Matthew R. Sydes, Sally Wilkes, Helen Nankervis, Shelley Dowey, Hazel Bell, Margaret Cruickshank, Karen Gibbon, Cathy M. Green, Christina Wong, Caroline M. Owen, Kate London, Shaheen Haque, Kim S. Thomas

**Affiliations:** 1 Centre of Evidence Based Dermatology, King's Meadow Campus, Lenton Lane, University of Nottingham, Nottingham, UK;; 2 Sheffield Teaching Hospitals NHS Foundation Trust, Sheffield, UK;; 3 MRC Clinical Trials Unit at UCL, Institute of Clinical Trials & Methodology, London, UK;; 4 Broadgreen Hospital, Royal Liverpool and Broadgreen University Hospitals NHS Trust, Liverpool, UK;; 5 Aberdeen Royal Infirmary, NHS Grampian, Aberdeenshire, UK;; 6 Whipps Cross University Hospital, Barts Health NHS Trust, London, UK;; 7 Ninewells Hospital & Medical School, NHS Tayside, Dundee, UK;; 8 Salford Royal Hospital, Salford Royal NHS Foundation Trust, Salford, UK;; 9 Royal Blackburn Hospital, East Lancashire Hospitals NHS Trust, East Lancashire, UK;; 10 St Luke's Hospital, Bradford Hospitals Foundation Trust, West Yorkshire, UK; and; 11 Addenbrookes Hospital, Cambridge Hospitals NHS Foundation Trust, Cambridge, UK

**Keywords:** vulvar, erosive lichen planus, systemic treatment, vulvovaginal, randomized controlled trial

The aim of this commentary is to document our experience and lessons learned of running a randomized controlled trial (RCT) in vulvar erosive lichen planus (ELPV), an uncommon and underresearched condition. Vulvar erosive lichen planus causes painful vulvovaginal erosions, which affect daily function and quality of life.^1^ Response to standard first-line therapy (superpotent topical corticosteroids) is often inadequate, and there are no RCTs to guide second-line treatment.^2^ The “hELP” (Systemic Therapy for Vulvar Erosive Lichen Planus) trial was a pilot study to assess feasibility of a definitive trial comparing systemic treatments for ELPV. Ethical approval (14/YH/0046), prospective trial registration (ISRCTN: 81883379), and protocol publication^3^ occurred.

“hELP” was a multicenter, four-arm, assessor-blind, pilot RCT recruiting from 12 UK sites for 14 months. Eligible participants were randomized to a 6-month course of hydroxychloroquine, methotrexate, or mycophenolate mofetil or a 4-week reducing regimen of prednisolone (comparator group); all received super-potent topical corticosteroids.

Inclusion criteria were the following: women older than 18 with a clinical diagnosis of moderate to severe ELPV, despite 3-month treatment with clobetasol propionate 0.05%, plus documented vulvar biopsy that excluded malignant/premalignant disease. Participants must have agreed to clinical photographs.

Exclusion criteria were the following: (1) lichen sclerosus/lichen planus overlap; (2) received any of the systemic trial drugs within the last month; (3) a previous or current diagnosis of malignant disease; (4) premalignant cervical or vulvar disease; (5) live vaccine administration in the last 2 weeks; (6) pregnancy or breastfeeding; (7) allergy to any of the trial medications; (8) history of clinically significant renal/liver impairment or concurrent medications that would interact with the trial drugs; and (9) any other reason that the trial medications would not be given in usual clinical practice.

Feasibility outcomes were the proportion of eligible participants randomized; the proportion of patients adhering to treatment, quality and suitability of clinical images, suitability of trial design, and suitability of clinical outcomes. The primary clinical outcome was treatment “success” at 6 months. Because of the absence of validated outcome measures for ELPV, the definition of “success” was agreed after qualitative work with expert clinicians.^4^ Treatment was classed as successful if both the following outcomes were met:

1. Patient global assessment of disease severity of “none” or “mild” (on a 4-point scale of none, mild, moderate, or severe disease).
2. Any improvement from baseline judged by blinded assessment of clinical photographs.

The trial was pragmatically designed; interventions were tested in an environment that was as close to real-life as possible in terms of setting, study population, intervention, comparator, and outcomes.^5^

**FIGURE 1:**
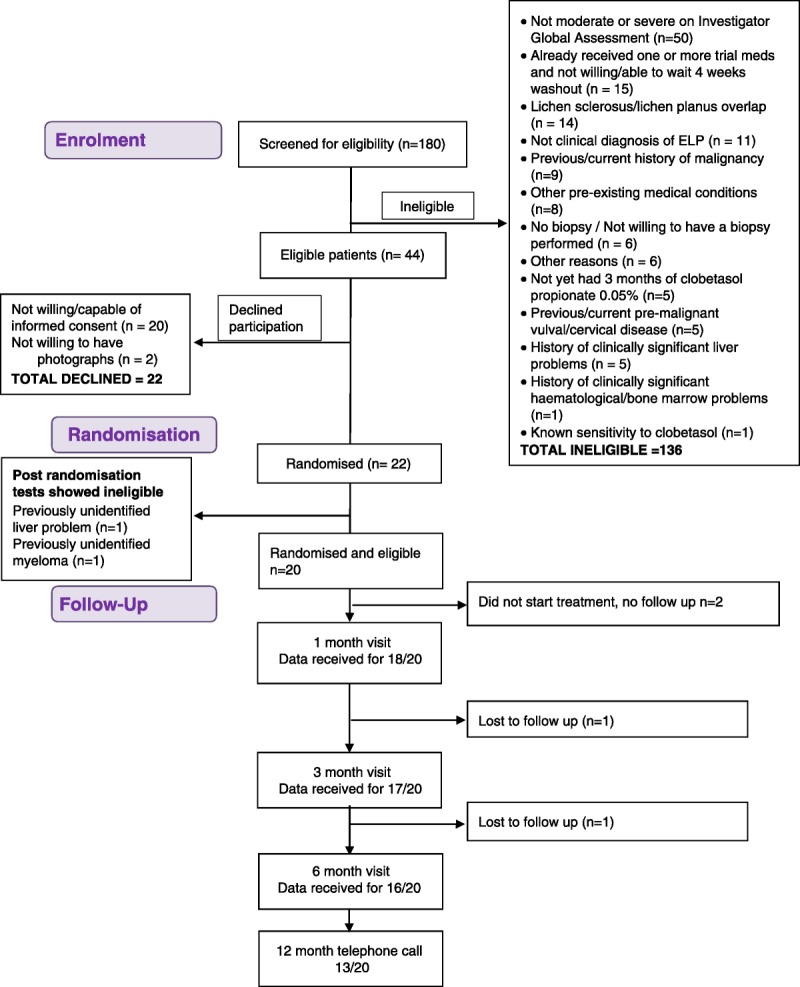
CONSORT diagram of participant flow in the hELP pilot trial.

Of 180 patients screened, only 44 (24%) were eligible. Ineligibility reasons are in Figure 1. Of those eligible, 22 (50%) of 44 were randomized; 20 did not consent to take a tablet treatment. The study was closed without reaching its recruitment target of 40. For those 22 patients who entered the trial, study medications were not started by four participants, four stopped trial treatment early and two were lost to follow-up (Figure 1).

Only 14 of 22 participants had complete before and after images, and overall quality was poor despite being taken by medical photography. Treatment “success” only occurred in the hydroxychloroquine (2/6, 33%) and mycophenolate mofetil (2/5, 40%) groups.

hELP was an ambitious trial because it was looking to recruit patients with an uncommon skin condition for second-line treatment. However, preliminary data had suggested that the recruitment target was achievable. The lessons learned are summarized in Table 1, and specific lessons learned for future ELPV trials are expanded upon hereinafter.

**TABLE 1:**
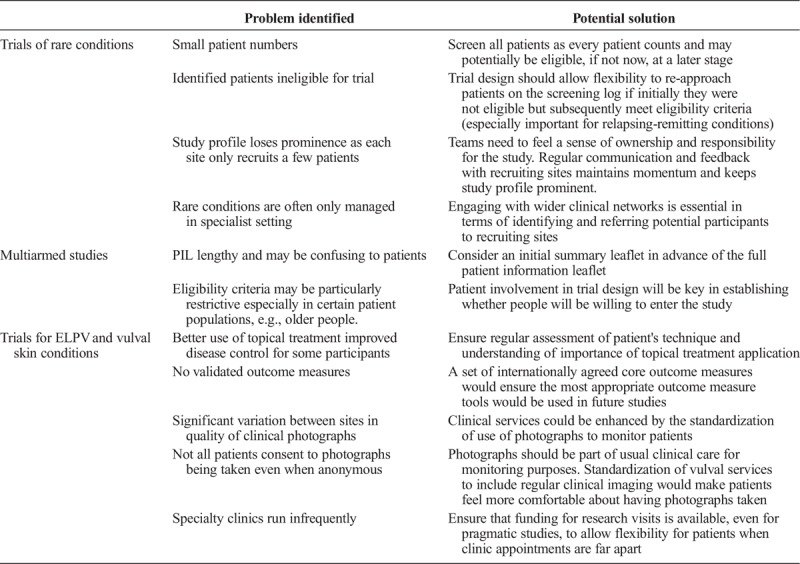
Lessons Learned From the hELP Study: The Lessons Learned Have Been Categorized and Potential Solutions Suggested to Inform Other Researchers' Trial Designs

Despite 180 patients being identified, many were not eligible. The main ineligibility reason was mild disease. In counseling potential participants for hELP, some were found to not be using topical steroids effectively; re-education on topical treatment, for some, led to better disease control and negated the need for systemic therapy. This suggests that over time, patients' technique of applying and understanding of the importance of topical treatments was lessening and not being regularly assessed by clinical care teams.

Of those eligible, the main reason for nonparticipation was that people did not want to take a tablet treatment, despite clinically moderate/severe disease. People with ELPV are often of an older age group, likely to have comorbidities and be more anxious about combining medications or adverse effects. This should be borne in mind for future trials as reluctance for systemic therapy may always present a barrier to recruitment.

Patient-reported symptoms are a guide for therapeutic decision-making^4^ and are arguably the most important outcome to measure. However, because this was an open-label trial, we wanted to ensure a blinded component to the clinical end point through using objective assessment of clinical photographs. Only four participants achieved our definition of “treatment success.” However, 10 of 16 showed improvement in patient global assessment and 6 of 16 continued treatment after 6 months. This suggests that the composite primary outcome was too stringent and a set of “core outcome measures”^6^ is essential to success of future ELPV trials.

Despite clinical photography being usual practice in the management of vulval disease, the concept of taking images deterred 2 (4%) of 44 of eligible patients from consenting to the study. In addition, 6 (46%) of 13 contacted at 12 months stated that they found the photographs embarrassing or that they were not keen on having them taken.

Photographs received were of varying quality, despite the provision of a standardized photographic protocol. Medical imaging provision varied widely between centers. Some had photographers present in the clinic who could take the images immediately. Others only operated at specific times, often in a different location within the hospital. The latter led to practical difficulty, especially if images needed to be retaken because of inadequate initial images.

Important lessons applicable to developing and delivering future trials have been learned by conducting the hELP study. Running a pilot trial was an important step to assess feasibility and in this case stopped a full-scale RCT from proceeding. Valuable resources have therefore been saved, and we hope that the lessons learned will prevent future research waste in other areas.
